# The spatiotemporal evolution of rural landscape patterns in Chinese metropolises under rapid urbanization

**DOI:** 10.1371/journal.pone.0301754

**Published:** 2024-05-06

**Authors:** Ninghan Xu, Peng Zeng, Yuanyuan Guo, Muhammad Amir Siddique, Jinxuan Li, Xiaotong Ren, Fengliang Tang, Ran Zhang

**Affiliations:** Department of Urban Planning, School of Architecture, Tianjin University, Tianjin, China; Van Lang University: Truong Dai hoc Van Lang, VIET NAM

## Abstract

Understanding the evolution of rural landscapes in metropolises during rapid urbanization is crucial for formulating policies to protect the rural ecological environment. In this study, remote sensing and geographical information system data, as well as applied landscape index analysis, are used to examine the spatiotemporal evolution of rural landscape patterns in the Beijing-Tianjin region of China, which has experienced rapid urbanization. The relationships between land use/land cover changes and changes in rural landscape patterns are explored. The results revealed significant spatial differences in the rural landscapes in the Beijing-Tianjin region; farmland and forestland were the main types of landscapes, creating a "mountain-field-sea" natural landscape pattern. The conversion of rural landscapes in the Beijing-Tianjin region involved mainly the conversion of farmland to urban areas, with few exchanges between other landscape types. The urban areas in the Beijing-Tianjin region increased by 3% per decade; farmland decreased at the same rate. Additionally, the rural landscape patterns in the Beijing-Tianjin region were dominated by fragmentation, dispersion, and heterogeneity and moved from complex to regular. Water bodies displayed the most fragmented natural landscape; their number of patches increased by 36%, though their network characteristics were maintained. Forestland was the most concentrated natural landscape. In this study, theoretical support and a scientific reference for the optimization of rural landscape patterns and the improvement in rural living environments in rapidly urbanizing areas are provided.

## Introduction

Rural areas in China span vast territories and occupy approximately 94% of the country’s total land area. Rural landscapes provide the natural foundation for rural life [1,2]; their quality is crucial for rural inhabitants [3,4]. During the past four decades, rapid urbanization in China has led to significant changes in land use in metropolises, which consequently impacts rural landscapes [5]. Accordingly, balancing rapid urban expansion with the quality of the living environment provided by rural landscapes has become a critical issue [6–8]. Typically, the pattern of rural landscapes is a manifestation of landscape heterogeneity [9,10]. Exploring the changes in rural landscape patterns influenced by rapid urbanization can provide reference value for the sustainable development of rural living environments [11–13].

Land use and land cover change (LUCC) directly affect landscape patterns [14], and landscape pattern changes are the most intuitive manifestations of LUCC [15–17]. At present, satellite remote sensing (RS) technology enables the monitoring of land use changes [18]. Remote sensing imagery provides an excellent data source from which land use change information can be effectively extracted, analyzes, and simulated [19,20]. Land use changes can be identified [21], and the characteristics, changes, and values of rural landscapes can be analyzed using remote sensing technology [22] and geographic information system (GIS) technology [23–25]. In recent years, LUCCs have gradually become the focus of research in geography and urban planning [26]. Existing studies have mainly considered the processes, trends, and driving factors of land use type changes [15,27] and have combined land use data to analyze the spatial change patterns of rural settlements [28–30]. As the study of land use change has evolved, land use has become the foundation of landscape pattern research.

Landscape pattern analysis, one research topic within the field of landscape ecology, is conducted in a two-dimensional plane, thus enabling the discovery of potential patterns [31] and constituent units or element combinations in widely distributed landscapes [32,33]. To obtain the landscape pattern features and spatial heterogeneity in regional landscape dynamics, landscape indices have been developed [2,34]. A landscape pattern index can not only quantitatively describe the dynamic changes in landscape patterns [35] but also clearly reflect the structural composition and spatial configuration of the landscape [36]. To date, landscape pattern indices have been widely applied in and are considered fundamental methods for landscape pattern analysis [37].

Research on landscape pattern changes has considered mainly dynamic change patterns and their driving factors [38], urban area functional landscape patterns [39,40], suburban area landscape pattern characteristics [41], spatiotemporal heterogeneity, and driving factor analysis of landscape fragmentation [42,43]. First, land use change has a direct impact on landscape patterns [24,44,45]. Landscape pattern changes have a significant relationship with ecological security, which is highly sensitive to landscape fragmentation [46]. In addition, landscape patterns significantly affect habitat quality, as the aggregation and compactness of landscape patterns are negatively correlated with habitat quality [47].

Urban expansion is a factor affecting landscape patterns [48]. In a rapidly urbanizing area, the new urban core area rapidly develops in areas distanced from the existing central urban area; this process leads to obvious fragmentation [49–51]. Existing urban areas continue to expand, causing an agglomeration of urban areas to develop during different periods of growth [52]. Studies have shown that the degree of land use diversity and landscape fragmentation are positively correlated with the degree of urbanization [53]. For instance, rapid urbanization can lead to spatiotemporal differences in agricultural spatial functions, resulting in urban area–rural gradient changes in agricultural spatial function combinations [54–56]. The study of LUCC has established methods for the identification, classification, and analysis of land units with clear boundaries and enabled landscape pattern research to be based on the ’patch-corridor-matrix’ model [41]. Research has revealed certain patterns of land use change resulting from urban expansion. However, under different policy influences and socio-economic development contexts, distinct urban expansion and landscape pattern change patterns emerge. China employs a collective land system and a socialist market economy. Under this unique institutional guidance, the government’s strong intervention capability has led to rapid population growth in rural areas surrounding metropolises, dramatic changes in land use types, and swift urbanization and rural transformation. This process has also resulted in varying degrees of rural landscape degradation. However, current research on the patterns of change in rural landscape patterns and their mechanisms in such areas remains relatively scarce. Therefore, this study not only focuses on the general impacts of urban expansion on rural landscape changes but more importantly, reveals how these impacts exhibit unique regional characteristics under China’s specific socio-economic and land policy backdrop.

The Beijing-Tianjin region is located at the center of the Beijing-Tianjin-Hebei urban agglomeration. This region is one of China’s seven major city clusters and serves as China’s political, cultural, and economic center. Simultaneously, the Beijing-Tianjin region, situated on the North China Plain, possesses a characteristic plain topography and is one of the cradles of ancient Chinese agricultural civilization. Since China’s reform and opening-up in 1978, the Beijing-Tianjin region has experienced rapid urbanization with rapid economic and population growth. Rapid urban expansion has led to the rapid deterioration of the ecological environment in surrounding rural areas. Studies have shown that the level of urbanization in the Beijing-Tianjin-Hebei region is negatively correlated with the evolution of vegetation cover [57]. Currently, an understanding of the characteristics of rural landscape pattern changes during the rapid urbanization process is urgently needed to curb the increasing deterioration of rural living environments [58] and to address the contradiction between urban expansion and rural environment protection [59]. Motivated by the above considerations, this study primarily explored the principles governing landscape pattern evolution in rural areas surrounding metropolises that are undergoing rapid urbanization. The objectives of this study were to 1) describe the spatial differentiation and characteristics of rural landscape patterns in the Beijing-Tianjin region and 2) quantitatively analyze and describe the changes in rural landscape patterns in the Beijing-Tianjin region between 1980 and 2018 and explore the relationship between LUCC and rural landscape pattern changes. This study provides theoretical support and scientific references for the optimization of rural landscape patterns and improvement in rural living environments in rapidly urbanizing areas.

## Materials and methods

### Study area

**Fig 1:**
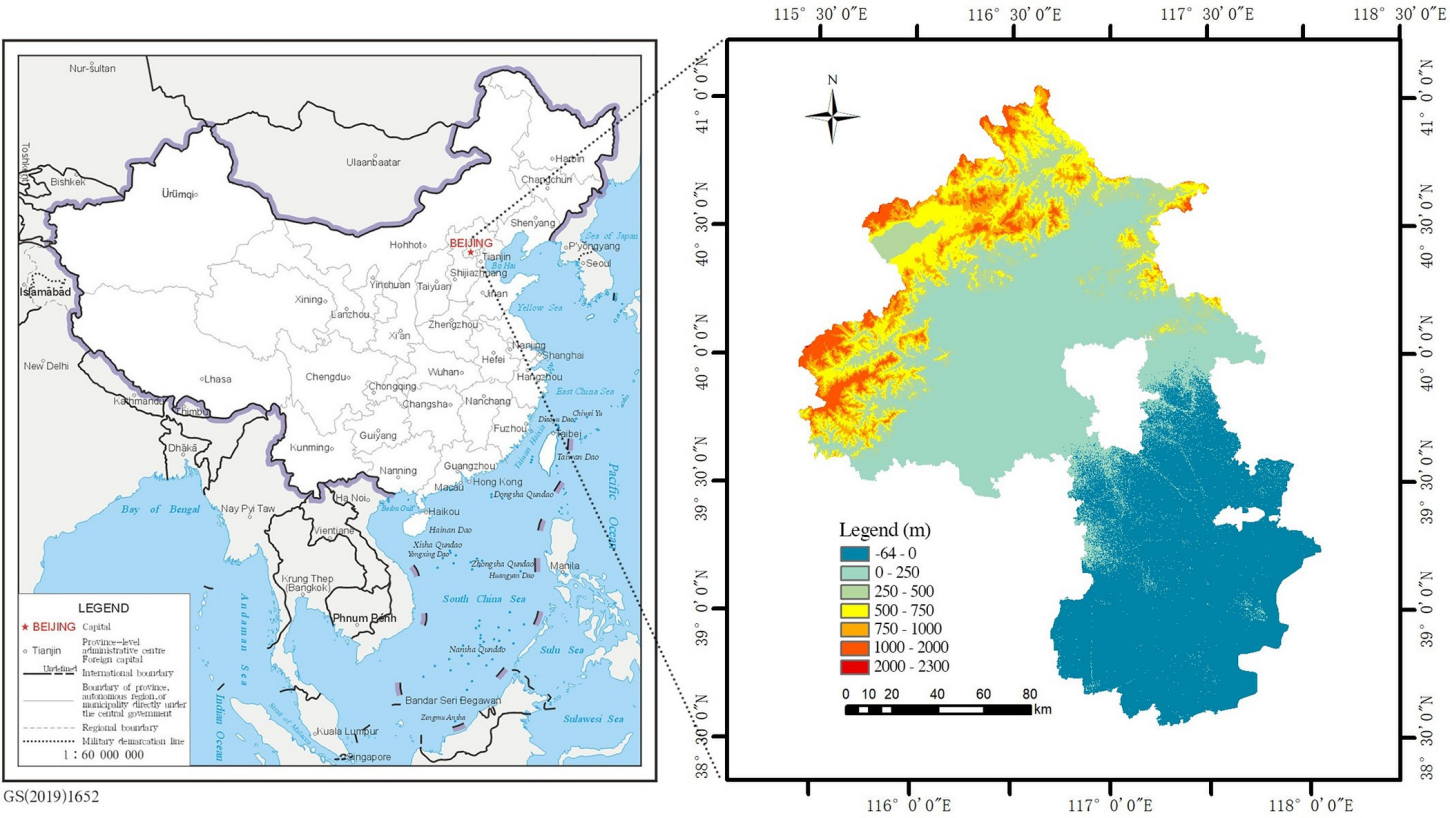
Geographical overview of the study area. The map on the left shows the location of the study area. The map on the right represents the digital elevation model (DEM) of the study area.

The Beijing-Tianjin region (38°34’E-41°03’E, 115°25’N-118°04’N) is located in the northern part of the North China Plain and consists of two major metropolises, Beijing and Tianjin (Fig 1). In 2018, the Beijing-Tianjin region had a land area of 28,376 km^2^ and a permanent population of 32.605 million people. Its terrain is more elevated in the northwest and less elevated in the southeast. The Beijing-Tianjin region has terrain features, including mountains, hills, plains, depressions, coasts, and tidal flats, as well as natural resources such as forests, grasslands, water bodies, lakes, and fields. The two cities are connected by railways, highways, and rivers. They also undertake close economic activities and experience population exchanges and biological mobility with one another. From 1980 to 2018, the urbanization rate of the Beijing-Tianjin region increased from 55.1% to 85.2%. Thus, during this period, the Beijing-Tianjin region experienced rapid urbanization.

### Data sources and processing

Since the implementation of the Reform and Opening-up Policy in 1978, China has entered a phase of rapid urbanization marked by significant changes in land use and substantial alterations in rural landscape patterns. Between 1980 and 2018, the urbanization rate in the Beijing-Tianjin area increased from 55.1% to 85.2%, revealing a rapid urbanization process. Therefore, this study utilized data from 1980 to 2018, which are representative of the evolution of rural landscape patterns during the rapid urbanization process.

In this paper, Landsat remote sensing imagery data from the U.S. is utilized (http://landsat.visibleearth.nasa.gov/). Through manual visual interpretation, a land use remote sensing monitoring database was established. The main information sources used were Landsat TM remote sensing images from 1980, 1990, and 2010; Landsat TM/ETM remote sensing images from 2000; and Landsat-8 OLI remote sensing images from 2018 for Beijing and Tianjin, with a spatial resolution of 30 m*30 m. The preprocessing of remote sensing images includes band extraction, false color synthesis, geometric precision correction, and image stitching and mosaicking by county. During the geometric precision correction process, the average positional error must be no more than 50 meters (two pixels). Subsequently, human-computer interaction for land use interpretation was conducted within the ArcMap 10.8 environment. Considering the quality of the remote sensing information in the study area, images from early May to mid-October were selected. The interpretation accuracy standard was set at 95% or higher for data collection on farmland and urban areas; 90% or higher for forestland, grassland, and water bodies; and 85% or higher for unused land. Considering the proportions of various landscapes and the "mountain-field-sea" landscape structure in the Beijing-Tianjin region, the primary objective of this study was to explore the changes in the four natural landscape elements of farmland, forestland, grassland, water bodies, and urban areas. Therefore, referring to the Chinese Academy of Sciences’ classification system for LULC [60], ArcGIS 10.0 was used to divide the LULC types of the study area into farmland, forestland, grassland, water bodies, urban areas, and unused land. In the land use classification, farmland includes paddy fields and dry land; forestland refers to areas used for forestry, including the growth of trees, shrubs, bamboo, and coastal mangroves; grassland refers to various types of grasslands with a coverage of more than 5%; water bodies include natural terrestrial waters and lands used for water conservancy facilities; urban areas encompass urban and rural settlements, industrial, mining, and transportation lands; and unused land refers to land that is currently not utilized. Finally, the LUCC raster data were imported into Fragstats 4.2, and the landscape index module was selected for analysis, thereby obtaining the landscape pattern index data for the study area.

### Research methods

#### Spatial pattern analysis via ArcGIS

ArcGIS 10.8 was used to perform spatial pattern analysis on the five batches of LUCC data from 1980 to 2018 in the Beijing-Tianjin region [61], generating landscape distribution maps and statistics on landscape patch information [62]. First, the Reclassify tool in the spatial analyst module was used to reclassify and adjust the display color blocks of the patches in the five periods of remote sensing image classification data according to farmland, forestland, grassland, water bodies, urban areas, and unused land [63]. Subsequently, the Zonal Geometry tool in the spatial analyst module was used to calculate the area, number of patches, and perimeter of various landscape types in the Beijing-Tianjin region throughout the five remote sensing imaging periods [64]. Finally, the Calculate Geometry tool was used to compile information on the area, direction, number of patches, and patch names of landscape transfers between different types, thus establishing a transition matrix. The land use transition matrix, an application of the Markov model to land use change, can not only quantitatively express the conversion between land use changes but also reveal the transfer rate between land use changes [45]. Generally, the Markov chain model is used to simulate transitions, parameters, and trends. Probability transition matrices were generated to predict and classify potential LUCC and urban development scenarios [5]. The formula is:

$$S_{ij}=[\begin{array}{ccc} S_{11} & \cdots & S_{1n}\\ \vdots & \ddots & \vdots \\ S_{n1} & \cdots & S_{nn} \end{array}]$$ (1)

where *S*_*ij*_ represents the landscape status between the initial and final periods of the study and *n* represents the number of landscape types. The vector represents the area of each landscape type and the composition of landscape structures at different periods.

#### Landscape pattern index analysis

A landscape index is a quantitative indicator that condenses landscape pattern information [65], reflecting the structural composition and spatial configuration of landscapes [45]. Landscape pattern indices can quantitatively display compositional characteristics [66], spatial configurations, and dynamic changes in land use [67–69]. LULC is one of the factors that influences landscape pattern change. Landscape pattern indices were selected to analyze the impact of LULC on landscape patterns [70]. Landscape pattern index analysis generally includes patch, class, and landscape indices at three scales [71,72]. Based on the land use change characteristics of the study area, in this study, the impact of land use change on landscape patterns at the class and landscape scales was analyzed. The following nine class metric indices were selected: class area (CA), percentage of landscape (PLAND), number of patches (NP), patch density (PD), largest patch index (LPI), mean patch area (AREA_MN), aggregation index (AI), patch cohesion index (COHESION), and mean patch fractal dimension (FRAC_MN) (Table 1). The indices were classified into five categories: scale, fragmentation, concentration, networking, and shape. The following seven landscape metric indices were selected: the splitting index (SPLIT), LPI, landscape shape index (LSI), contagion (CONTAG), landscape division index (DIVISION), Shannon’s evenness index (SHEI), and Shannon’s diversity index (SHDI). The genes were classified into four categories: fragmentation, shape, connectivity, and heterogeneity [71] (Table 1). These 16 indices were used to analyze the impact of land use change in the Beijing-Tianjin region on landscape patterns. In other studies, scholars have used a single landscape index to characterize landscape patterns, which may introduce some errors into the results. In this study, multiple landscape indices were employed to represent a landscape pattern, reducing the possibility of errors and making the research results more convincing. However, landscape index analysis still has several shortcomings. For example, it can explain the degree of landscape fragmentation only by comparing the sizes of two or more SPLIT indices; it cannot directly assess the fragmentation level of a landscape through a single SPLIT index. Landscape pattern indices were calculated using Fragstats 4.2 [72]; the class metrics module was used to calculate the landscape indices at the class level; and the patch metrics module was used to calculate the landscape indices at the landscape level.

**Table 1:** Landscape pattern indices used in the study [72].

Index analysis	Characterization category	Index	Definition	Formula
Class metric indices	Scale	CA	Reflects landscape type patch area.	$CA=\sum_{j=1}^{n}a_{ij}(\frac{1}{10000})$
		PLAND	Sum of the areas of all patches, divided by total landscape area.	$PLAND=P_{i}=\frac{\sum_{j=1}^{n}a_{ij}}{A}(100)$
	Fragmentation	NP	Number of patches divided by area.	$NP=n_{i}$
		PD	Number of patches divided by total.	$PD=\frac{n_{i}}{A}(10000)(100)$
		LPI	Reflects the proportion of the largest patch of a landscape type to the total landscape area, this metric aids in determining landscape scale or dominant types.	$LPI=\frac{\begin{array}{c} n\\ \max\\ j=1 \end{array}\left(a_{ij}\right)}{A}(100)$
		AREA_MN	Reflects the average patch area and landscape fragmentation degree.	$AREA\_MN=\frac{\sum_{i=1}^{n}a_{ij}}{n_{j}}(\frac{1}{10000})$
	Concentration	AI	Reflects the nonrandomness or degree of aggregation of different patch types within a landscape.	$AI=[\frac{g_{ii}}{max\to g_{ii}}](100)$
	Networking	COHESION	Reflects the connectivity of landscape patches.	$COHESION=\left[1-\frac{\sum_{j=1}^{m}p_{ij}}{\sum_{j=1}^{m}p_{ij}\sqrt{a_{ij}}}\right]{\left[1-\frac{1}{\sqrt{A}}\right]}^{-1}\times 100$
	Shape	FRAC_MN	Reflects the shape of the landscape, with the value being positively correlated to the complexity of the shape.	$FRAC\_MN=\sum_{i=1}^{m}\sum_{j=1}^{n}2\ln\left(\frac{0.25p_{ij}}{\ln a_{ij}}\right)/N$
Landscape metric indices	Fragmentation	SPLIT	Reflects the degree of landscape fragmentation.	$SPLIT=\frac{A^{2}}{\sum_{i=1}^{m}\sum_{j=1}^{n}a_{ij}^{2}}$
		LPI	Reflects the proportion of the largest patch of a landscape type relative to the entire landscape area.	$LPI=\frac{max(a_{ij})}{A}(100)$
	Shape	LSI	Reflects landscape shape, with the value being positively correlated to the complexity of the shape.	$LSI=\frac{0.25E^{*}}{\sqrt{A}}$
	Connectivity	CONTAG	Measures the extent to which patch types are aggregated or clumped.	$CONTAG=\left[1+\frac{\sum_{i=1}^{m}\sum_{k=1}^{m}\left[P_{i}\frac{g_{ik}}{\sum_{k=1}^{m}g_{ik}}][\ln\left(P_{i}\frac{g_{ik}}{\sum_{k=1}^{m}g_{ik}}\right)\right]}{2\ln\left(m\right)}\right](100)$
		DIVISION	Division is based on the cumulative patch area distribution and is interpreted as the probability that two randomly chosen pixels in the landscape are not situated in the same patch.	$DIVISION=\left[1-\sum_{i=1}^{m}\sum_{j=1}^{n}{\left(\frac{a_{ij}}{A}\right)}^{2}\right]$
	Heterogeneity	SHEI	Reflects the degree of unevenness in the distribution of patch areas within the landscape.	$SHEI=\frac{-\sum_{i=1}^{m}(P_{i}\ln P_{i})}{\ln m}$
		SHDI	Reflects the complexity and variability of different patch types within the landscape.	$SHDI=-\sum_{i=1}^{m}(P_{i}\ln P_{i})$

^1^ *aij* = area (m) of patch *ij*; *Pi* = proportion of the landscape occupied by patch type (class) *i*; A = total landscape area (m2); *gii* = number of like adjacencies (joins) between pixels of patch type (class) *i* based on the single-count method; *max-gii* = maximum number of like adjacencies (joins) between pixels of patch type (class) *i* (see below) based on the single-count method; *e ik* = total length (m) of edge in landscape between patch types * (classes) *i* and *k*; includes the entire landscape boundary and some or all background edge segments involving class *i*; *pij* = perimeter of patch *ij* in * terms of number of cell surfaces; *Z* = total number of cells in the landscape; *ni* = number of patches in the landscape of patch type (class) *i*; *gik* = number of adjacencies (joins) between pixels of patch types (classes) *i* and *k* based on the double-count method; *m* = number of patch types (classes) present in the landscape, including the landscape border if present; E* = total length (m) of edge in landscape includes the entire landscape.

## Results

### Spatial differentiation and characteristics of rural landscapes in the Beijing-Tianjin region

#### Spatial differentiation of rural landscapes

**Fig 2:**
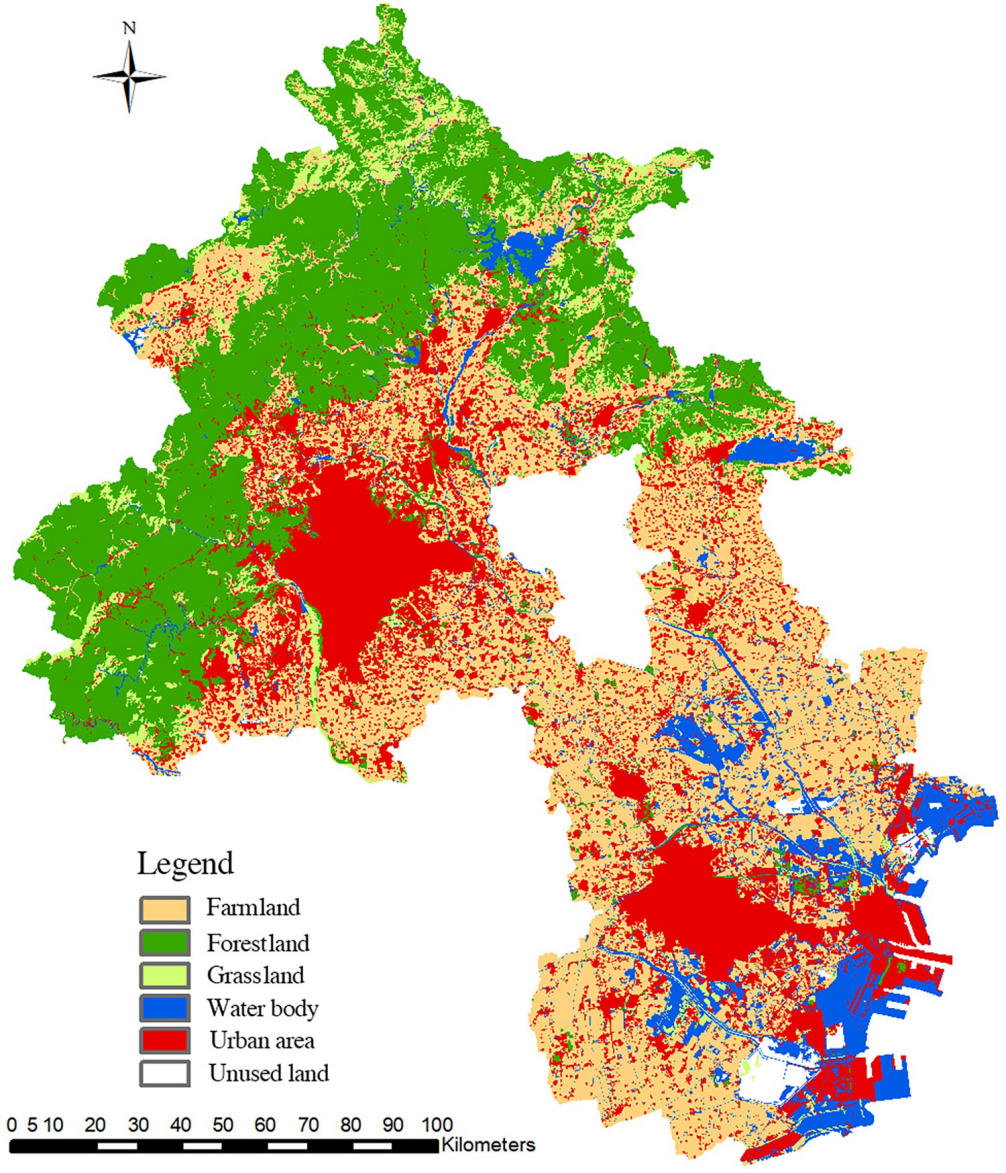
Distribution map of rural landscapes in the Beijing-Tianjin region in 2018.

**Fig 3:**
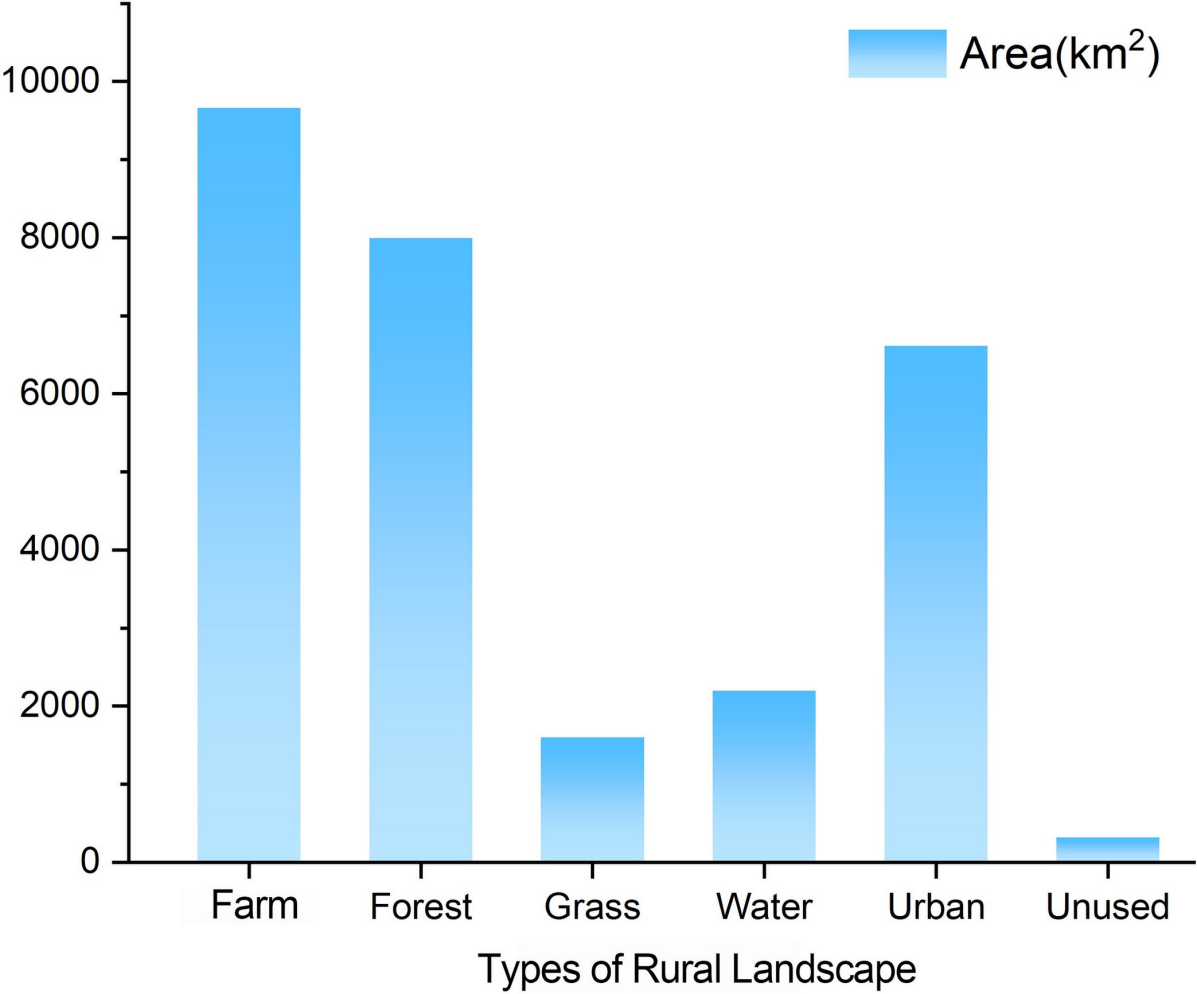
Areas of various rural landscape types in Beijing and Tianjin in 2018.

According to the distribution of natural rural landscapes in the Beijing-Tianjin region in 2018 (Fig 2), farmland (9600.58 km^2^) is the dominant landscape type and accounts for the largest proportion of the landscape in the region; it is distributed mainly in the central plain areas and is the primary landscape type surrounding the cities (Fig 3). The second most abundant landscape type is forestland (7964.15 km^2^), which is predominantly located in the mountainous areas of the northwest. Grassland (1593.96 km^2^) and water bodies (2113.82 km^2^) are relatively scarce. Grasslands are mainly distributed in the mountainous areas of the northwest and are blended with forestlands. Water bodies are primarily found in southeastern Tianjin. Overall, the Beijing-Tianjin region has a large area of farmland; however, the area of water bodies is relatively small, placing considerable pressure on the water supply for agricultural production. This situation has caused the Beijing-Tianjin region to be one of the most water-scarce regions in China in the long term. Consequently, the spatial differentiation of natural rural landscapes in the Beijing-Tianjin region is significant, with forestland and grassland concentrated in the northwestern mountainous areas. The water bodies originate from northwestern mountainous areas, flow through central villages and farmland, converge in the southeast, form water systems, and eventually enter the Bohai Sea, creating a "mountain-field-sea" natural landscape pattern.

The integrity of this type of natural landscape pattern is relatively high, which is conducive to the formation of relatively clear ecological functional zoning. The northwestern region, where forestland and grassland are concentrated, is suitable as an ecological conservation hinterland for the Beijing-Tianjin region. The southeastern region, which is scattered with water bodies, is suitable for agricultural production, trade and transportation development, and population movement. The central plain, where farmland is concentrated, is suitable for the development of large-scale agriculture and urban construction. However, this natural landscape pattern also has detrimental effects on the formation of landscape features. The high spatial differentiation of natural rural landscape types is not balanced overall, leading to a homogenization of natural rural landscape types around villages, with the distinctive features of the Beijing-Tianjin region’s rural landscape being not particularly evident.

#### Spatial characteristics of rural landscapes

**Fig 4:**
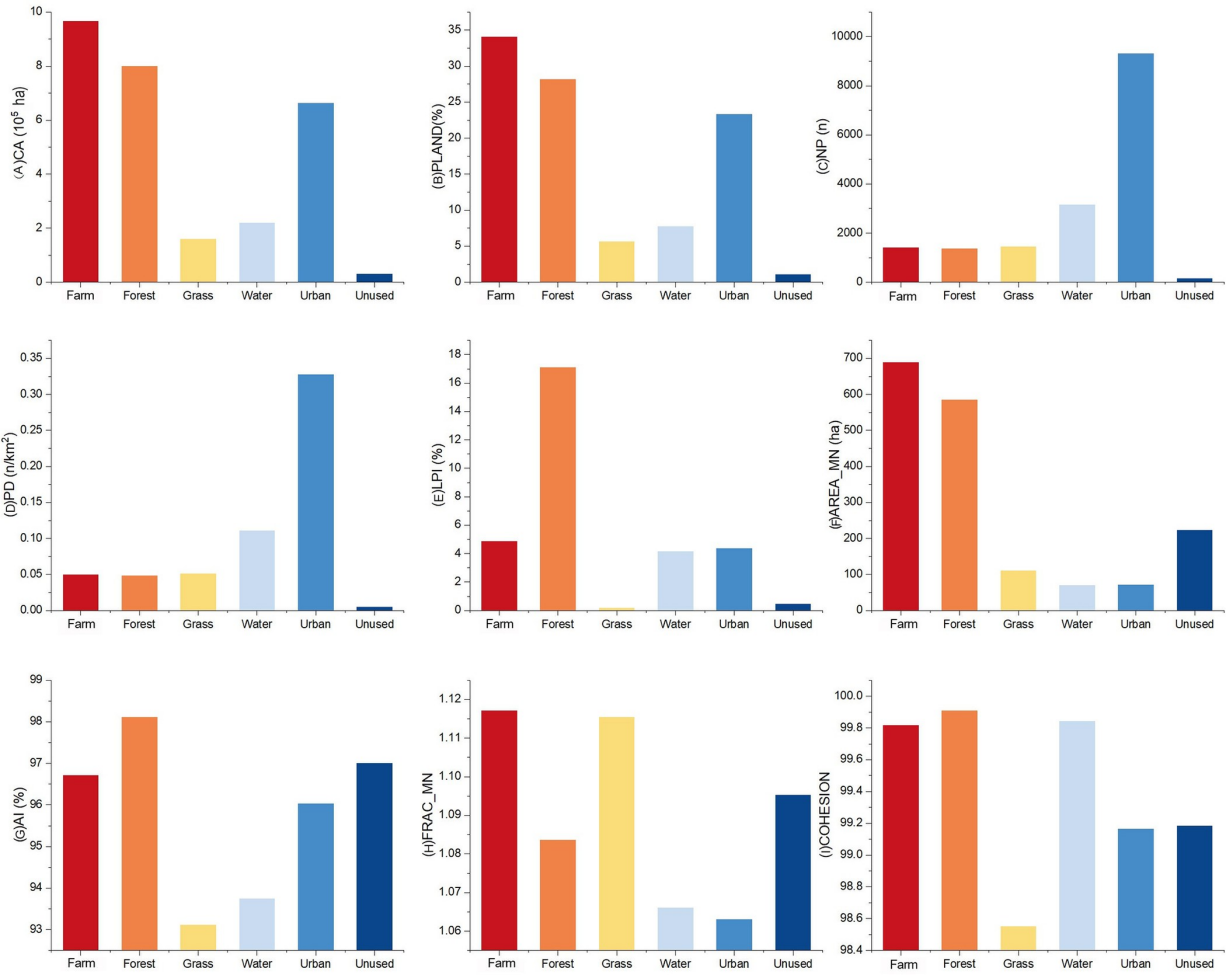
Landscape indices of various landscapes in the Beijing-Tianjin region in 2018 at the class level.

Fig 4 displays the landscape indices of the various landscape types in the Beijing-Tianjin region in 2018 at the class level. According to the classification in Table 1, the nine class indices express the four spatial characteristics of natural landscapes: scale, distribution, networking, and shape.

The scale characteristics can be characterized by area and distribution. As primary natural landscapes, farmland and forestland have relatively greater AREA_MN (Fig 4F) and PLAND (Fig 4B) values, indicating that farmland and forestland are distributed in large and numerous patches. The AREA_MN of grassland, which occupies a smaller proportion, is far smaller than that of farmland and forestland, whereas the NP of grassland is on par with that of farmland and forestland. This finding suggests that grasslands are distributed in smaller patches. A comparison of the class indices of the four types of natural rural landscapes reveals that water bodies have the highest number and density, while the number and density of the other three types of natural rural landscapes are relatively even.

Distribution characteristics can be measured by landscape fragmentation and concentration. The NP (Fig 4C) and PD (Fig 4D) of the water bodies are significantly greater than those of the other three types of natural landscapes, indicating that the water bodies have the highest fragmentation. Forestland, as the second largest natural landscape type in the Beijing-Tianjin region, has a much greater LPI (Fig 4E) than do the other three types of natural landscapes. However, its NP and PD are not prominent, indicating that forestland has the lowest fragmentation and presence of larger contiguous areas. As a less abundant natural landscape, grassland has a similar NP and PD but lower LPI than farmland, indicating that the fragmentation of grassland is greater than that of farmland. Therefore, the order of natural landscape fragmentation is water bodies > grassland > farmland > forestland. The AI (Fig 4G) of forestland is greater than that of the other three types of natural landscapes, indicating that forestland has the most apparent concentration. The concentration of farmland is also high, whereas that of water bodies and grassland is lower. Thus, in terms of natural landscape concentration, forestland > farmland > waterbody > grassland.

The networking characteristics can be characterized by the connectivity and aggregation degree of landscape patches. Water bodies have a much greater NP than do the other three types of natural landscapes; however, the AI is relatively low, and the COHESION (Fig 4I) is similar to that of the other three types of natural landscapes. This finding indicates that the water body patches are numerous, dispersed, and highly connected, with the highest degree of network structure. In comparison, while the connectivity of farmland, forestland, and grassland is also relatively high, their NP and aggregation degrees are relatively average; therefore, the higher connectivity is more likely due to the clustering of patches, and their networking features are not explicitly expressed.

The shape characteristics can be described by FRAC_MN. The FRAC_MN (Fig 4H) of both farmland and grassland are greater than those of forestland and water bodies, indicating that the shapes of forestland and grassland are more complex. The FRAC_MNs of forestland and water bodies are relatively close. Therefore, in terms of current characteristics, farmland > grassland > forestland ≈ water body.

### Evolution of rural landscapes

#### Dynamic changes in rural landscapes

**Fig 5:**
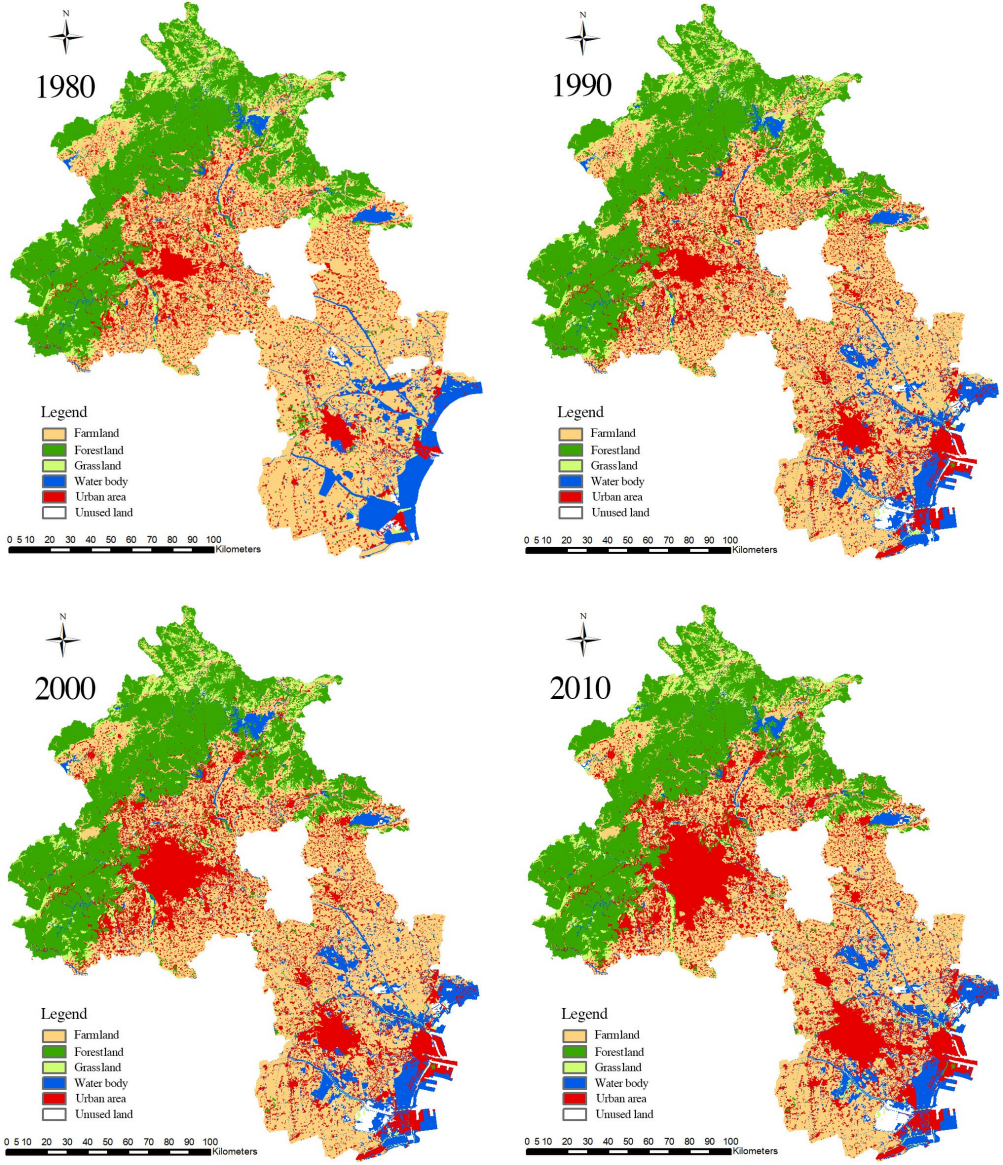
Distribution map of rural landscapes in the Beijing-Tianjin region from 1980 to 2018.

**Fig 6:**
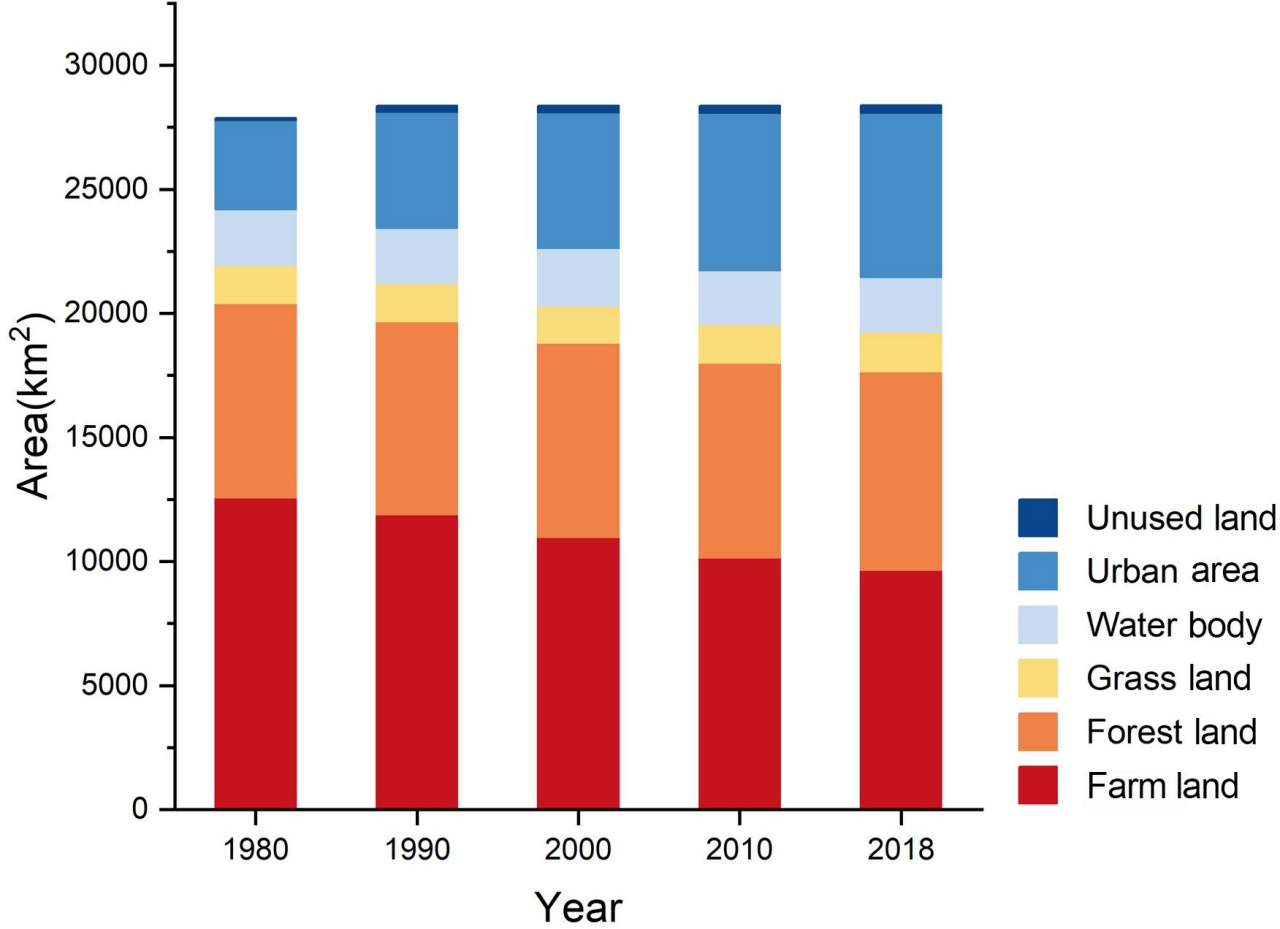
Areas of various rural landscapes in the Beijing-Tianjin region from 1980 to 2018.

Based on the distribution map (Fig 5) and statistical data (Fig 6) of the rural landscape in the Beijing-Tianjin region for five periods, the area and proportion of each type of landscape are as follows: farmland (34–45%) > forestland (27–28%) > urban area (13–23%) > water body (8%) > grassland (5–6%). Owing to the small proportion of unused land, it was not included in the analysis. Between 1980 and 2018, the most significant change in the natural rural landscape was the decrease in farmland, from 12,572.95 km^2^ in 1980 to 9,661.95 km^2^ in 2018, a total reduction of 2,911 km^2^, with an average annual decrease of 76.61 km^2^. The urban areas increased the most, from 3,592.53 km^2^ in 1980 to 6,615.55 km^2^ in 2018, with an average annual increase of 79.55 km^2^. Forestland, grassland, and water bodies did not change significantly. In the past four decades, the landscape types in the Beijing-Tianjin region have changed at a rate of a 3% decrease in farmland and a 3% increase in urban areas every ten years.

Forestland decreased by 46.38 km^2^ between 1980 and 1990 (Fig 6) and then continued to increase slightly, for a total of 209.15 km^2^, from 1990 to 2018. Grassland decreased by 71.29 km^2^ between 1980 and 2000 and then increased by 127.66 km^2^ between 2000 and 2018. Water bodies increased by 102.02 km^2^ between 1980 and 2000, peaked in 2000, and then continued to decrease, decreasing by 139.41 km^2^ between 2000 and 2018.

Overall, the farmland in the central part of the Beijing-Tianjin region continues to decrease, while the forestland and grassland in the northwestern mountainous areas show a slight upward trend, and the water bodies in the southeastern part show a slight downward trend.

An examination of the land use change maps of the five periods (Fig 5) indicates that rapid urbanization between 1980 and 2010 brought significant changes to the landscape structure in the Beijing-Tianjin region. First, urban areas expanded in a circular pattern around urban centers and rural residential areas, causing farmland to shrink and fragment. From 1980 to 2018, with rapid urbanization, the urban areas in the Beijing-Tianjin region continued to expand, while farmland continued to shrink. As shown in the distribution maps of rural landscape types in the Beijing-Tianjin region from 1980 to 2018, urban areas expanded in a circular pattern around urban centers and rural residential areas and were mainly centered on the urban areas of Beijing, Tianjin, and the Binhai New Area. The expansion of urban areas was rapid between 1980 and 2010 and then slowed after 2010. Among these periods, the expansion of urban areas was greatest between 1990 and 2000, during which time these areas exhibited a patchy distribution and began to gather in central urban areas and towns, gradually forming a network pattern. Due to geographic characteristics, there is a large area of farmland surrounding the construction areas in the Beijing-Tianjin region, providing conditions for urban area expansion. Therefore, as urban areas have expanded in a circular pattern around three central points and scattered rural residential areas, farmland has shrunk in a circular pattern, with whole blocks of farmland becoming fragmented. Second, while a small number of water bodies have been eroded during urban area expansion, their overall area has increased, and the overall network structure has been strengthened. Third, forestland and grassland, which are concentrated in northwestern mountainous areas, far from urban areas and rural residential areas, have been less affected by human activities, with no significant changes in area or spatial structure.

#### Conversion of different landscapes

**Fig 7:**
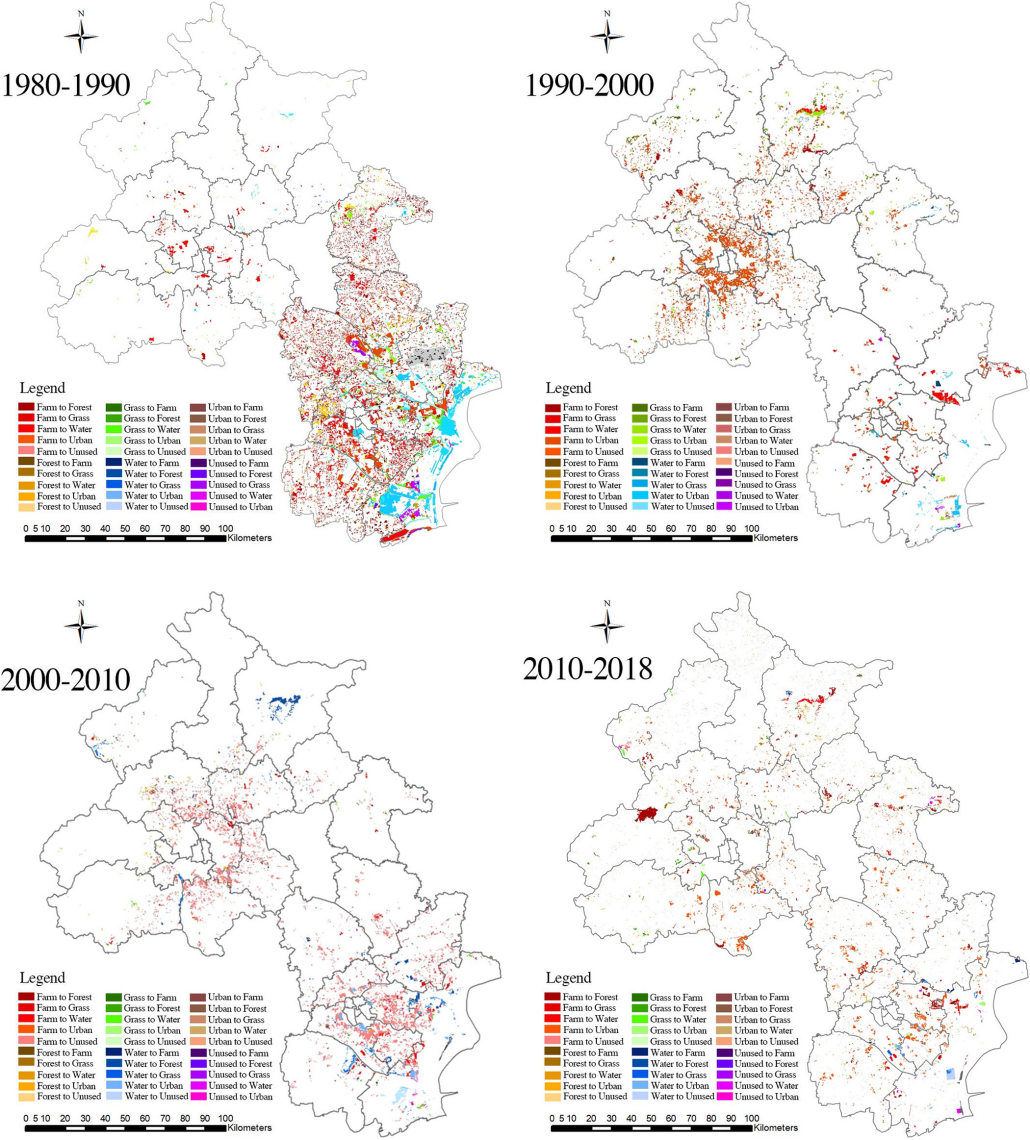
Spatial distribution of rural landscapes under conversion in the Beijing-Tianjin region from 1980 to 2018.

**Fig 8:**
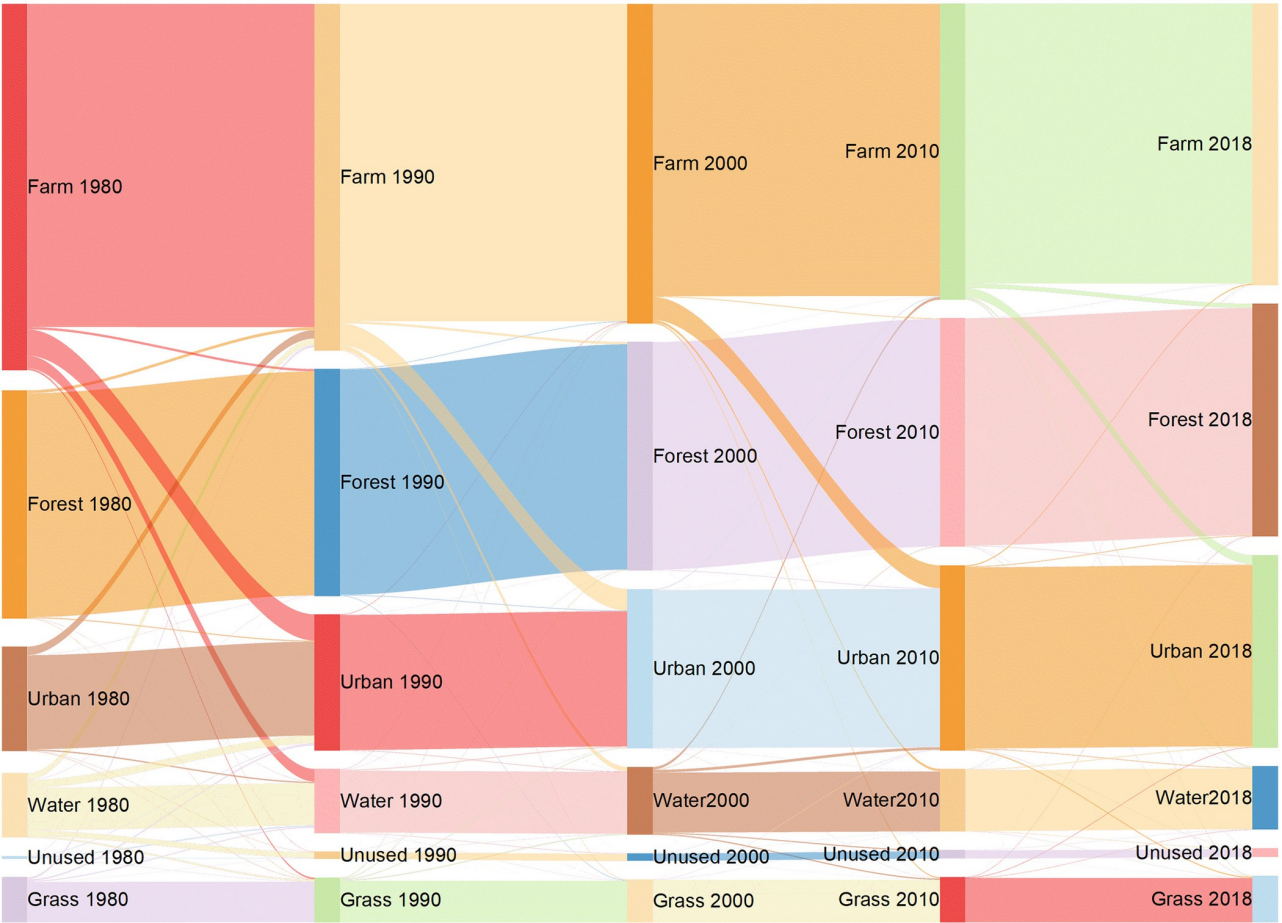
Conversion of rural landscapes in the Beijing-Tianjin region from 1980 to 2018.

The conversion to urban areas was greatest during 1980–1990 (Fig 7), the primary source of which was farmland, accounting for 903.67 km^2^ (Fig 8). The second-largest source was water bodies, accounting for 269.55 km^2^. Forestland and grassland were not significant sources of land for this conversion. Additionally, 435.80 km^2^ of farmland was converted to water bodies, and 292.27 km^2^ of water bodies was converted to farmland. There was noticeable two-way land conversion between farmland and water bodies, while forestland and grassland showed little tendency to convert to other landscape types.

Between 1990 and 2000, the largest increase in urban areas was primarily derived from farmland, whereas the conversion from other rural landscape types was relatively limited (Fig 8). During this period, farmland was primarily converted into urban areas (747.11 km^2^). This conversion amounted to 156.56 km^2^, which was less than the conversion in the previous period, corresponding to a 17% decrease in the total farmland area compared to that in the previous period. Water bodies were mainly converted into urban areas and farmland, with conversion areas of 37.37 km^2^ and 21.39 km^2^, respectively, which were significantly less than those in the previous decade. From the perspective of conversion to rural landscapes, the most prominent increase was in water bodies, with the primary source being farmland (163.70 km^2^). Overall, the transfer of forestland and grassland remained relatively stable, with minor fluctuations occurring between 1990 and 2000, while the area of farmland and water bodies converted to nonnatural landscapes decreased. Moreover, the conversion to water bodies increased, indicating that the protection of natural landscapes was strengthened during this decade and yielded noticeable results.

Between 2000 and 2010, the number of natural landscapes that were converted to nonnatural landscapes increased slightly (Fig 8). Among the converted natural landscapes, the main source was farmland, while the other three types of natural landscapes experienced relatively small shifts. Farmland was primarily converted into urban areas (788.7 km^2^); the conversion rate was 5.6% higher compared to that in the previous decade. Water bodies were converted mainly into urban areas and farmland, with conversion amounts of 92.8 km^2^ and 90.9 km^2^, respectively, an increase compared to the previous decade. This situation indicates that farmland will continue to be converted into urban areas and that natural landscapes will not be well protected.

Between 2010 and 2018, the area of farmland converted into nonnatural landscapes continued to decrease, with a more significant decrease and a more apparent continuous downward trend (Fig 8). Among the natural landscapes experiencing conversion, farmland remained the primary source, with a converted area of 320.21 km^2^ and a decrease of 426.90 km^2^ or 57% compared to that in the previous decade. The second direction of farmland conversion was toward forestland, with 150.23 km^2^ converted during this decade, an increase of 76.35 km^2^ or 103% compared to the previous decade. The conversions of water bodies, forestland, and grassland were relatively small. During this decade, the Beijing-Tianjin region strengthened its emphasis on the natural environment, increased efforts to protect natural landscapes, and achieved certain results in returning farmland to forestland and grassland. For example, ecological and economic forests were planted, and the urban area stock was reduced. In plain areas such as the Beijing-Tianjin region, farmland was continuously converted into urban areas through urbanization; however, and as urbanization gradually progressed, the transfer volume of farmland decreased.

### Evolution of landscape patterns in the Beijing-Tianjin region

#### Landscape pattern changes according to class metrics

**Fig 9:**
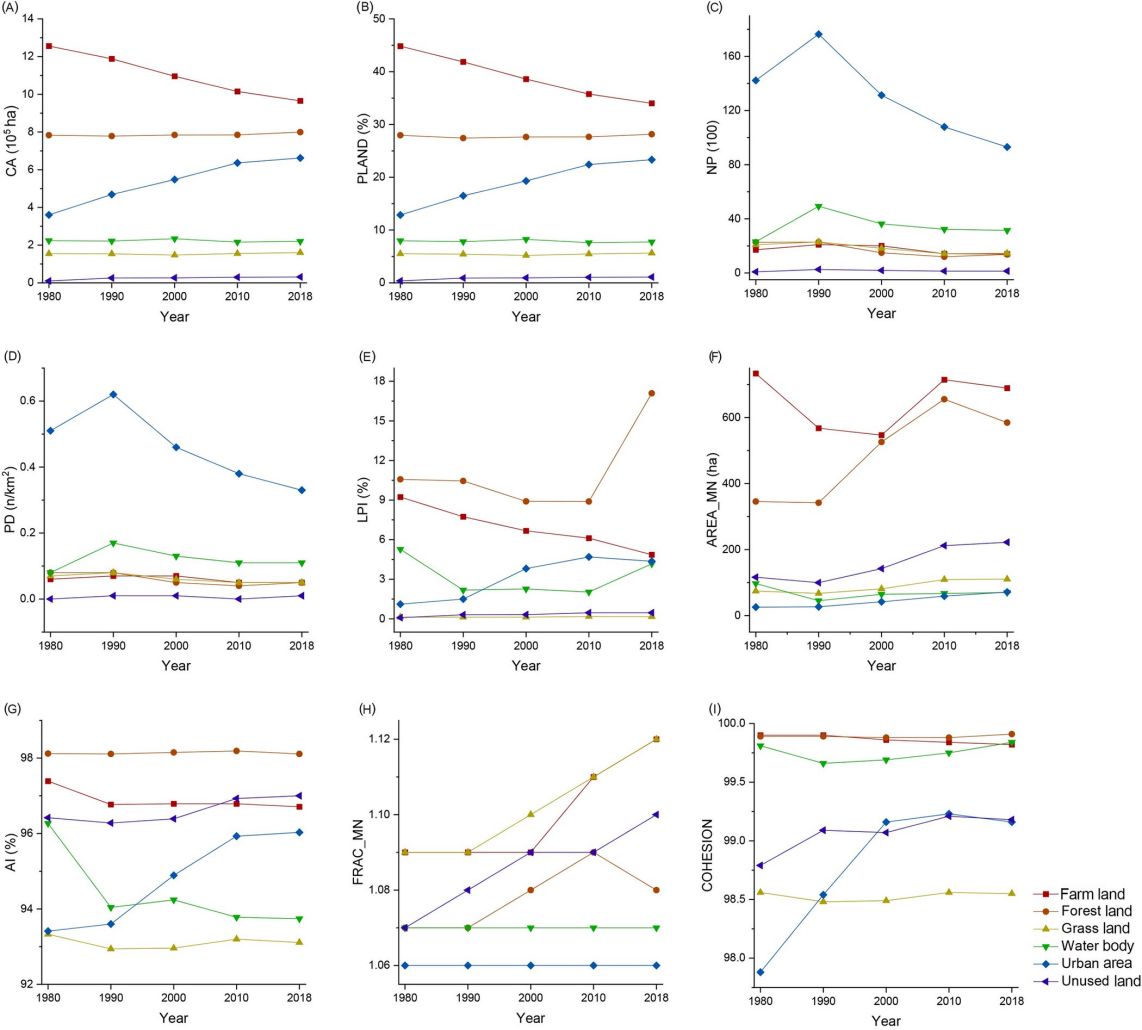
Changes in landscape pattern indices at the class level in the Beijing-Tianjin region.

According to Fig 9, between 1980 and 2018, the CA (Fig 9A) of farmland in the Beijing-Tianjin region continued to decrease, with an average annual decrease of 7651.89 km^2^, indicating a continuous reduction in the scale of farmland. However, under the trend of total quantity reduction, the NP (Fig 9C) and PD (Fig 9D) values of farmland first increased sharply and then decreased sharply, whereas the AREA_MN (Fig 9F) value first decreased slightly and then increased significantly, while the changes in AI (Fig 9G) were not significant. Farmland became more dispersed and fragmented between 1980 and 2000, after which it was gradually converted into other landscape types and became centralized. Between 1980 and 2018, the NP of the agricultural landscape initially increased and then decreased, while the COHESION (Fig 9I) and AI remained relatively stable. This situation indicates that there was no significant change in the degree of networked farmland and that patch distribution continued to be dispersed. According to the FRAC_MN (Fig 9H), the shape of farmland developed toward complexity and irregularity during 1980–1990 but improved between 1990 and 2018, gradually returning to a more regularized trend.

During 1980–1990, the forestland area (CA) decreased by 4643.64 km^2^, and the PLAND (Fig 9B) decreased from 27.95% to 27.42%. After 1990, the forestland area continued to increase, with an average annual increase of 746.73 km^2^, and its landscape proportion also rose from 27.42% to 28.16%, indicating that the scale of forestland experienced an initial contraction followed by expansion. Based on the LPI (Fig 9E), PD, and NP, the fragmentation of forestland remained stable during 1980–1990 and improved after 1990. The AI remained relatively stable. However, AREA_MN increased from 341.71 to 655.69 between 1990 and 2010 and then decreased to 584.74. Overall, the concentration of forestland was consistently strengthened, while the aggregation of patches decreased between 2010 and 2018, resulting in the disruption of landscape integrity. The COHESION and AI of forestland did not change significantly, indicating that the degree of networked forestland remained relatively unchanged. FRAC_MN showed no substantial changes, suggesting that the overall shape of the forestland remained stable.

The results for grassland area (CA) indicate that grassland experienced a process of contraction followed by expansion, with degradation occurring before 2000 followed by subsequent restoration (Fig 9A). Between 1980 and 1990, the PD and NP of the grassland increased slightly, while the AREA_MN decreased. After 1990, PD and NP continued to decline, whereas AREA_MN increased consistently. These findings suggest that from 1980 to 1990, grasslands exhibited a decrease in patch area, an increase in patch number and density, greater landscape fragmentation, and environmental degradation. Conversely, from 1990 to 2018, grassland area increased, patch number and density decreased, landscape fragmentation decreased, and environmental quality improved. The changes in the AI and LPI indicate that the concentration of grassland increased, as the aggregation degree of the patches increased from 1980 to 2010. Although grasslands became slightly dispersed afterward, this change did not affect the overall landscape concentration trend. The COHESION and AI of the grasslands did not change significantly, whereas the NP underwent an initial increase followed by a decrease. These results suggest that the network degree of grassland has not changed noticeably and is generally distributed in a relatively dispersed state. The sustained increase in FRAC_MN indicates that the shape of the grassland has been continuously tending toward complexity and irregularity, with this trend being more pronounced between 2010 and 2018.

The CA of the water bodies indicates that they underwent expansion followed by contraction (Fig 9A), maintained a favorable development trend before 2000, and then experienced degradation, with the scale in 2018 being smaller than that in 1980. The NPs of the water bodies increased substantially from 1980 to 1990, whereas the LPIs and AREA_MNs decreased significantly, and the total area and PD remained stable. The results suggest that the fragmentation of water bodies increased dramatically between 1980 and 1990, with water bodies being divided and landscape integrity decreasing. After 1990, the number and density of water body patches continued to decline, whereas the average patch area continued to increase, indicating that the trend of water body patches being damaged was curbed during this period, but the overall landscape quality was not restored.

The AI and PD of the water bodies continued to decline, suggesting that the aggregation of water bodies has been continuously decreasing, and patches have difficulty aggregating within a short time after being divided. The COHESION of water bodies decreased between 1980 and 1990 and then maintained an upward trend. The NP underwent an initial increase followed by a decrease. The results indicate that the network degree of water bodies declined before 1990, with the landscape developing toward a more dispersed trend. After 1990, the situation improved, with the distribution tending toward a networked trend.

#### Changes in rural landscape patterns according to landscape metrics

**Fig 10:**
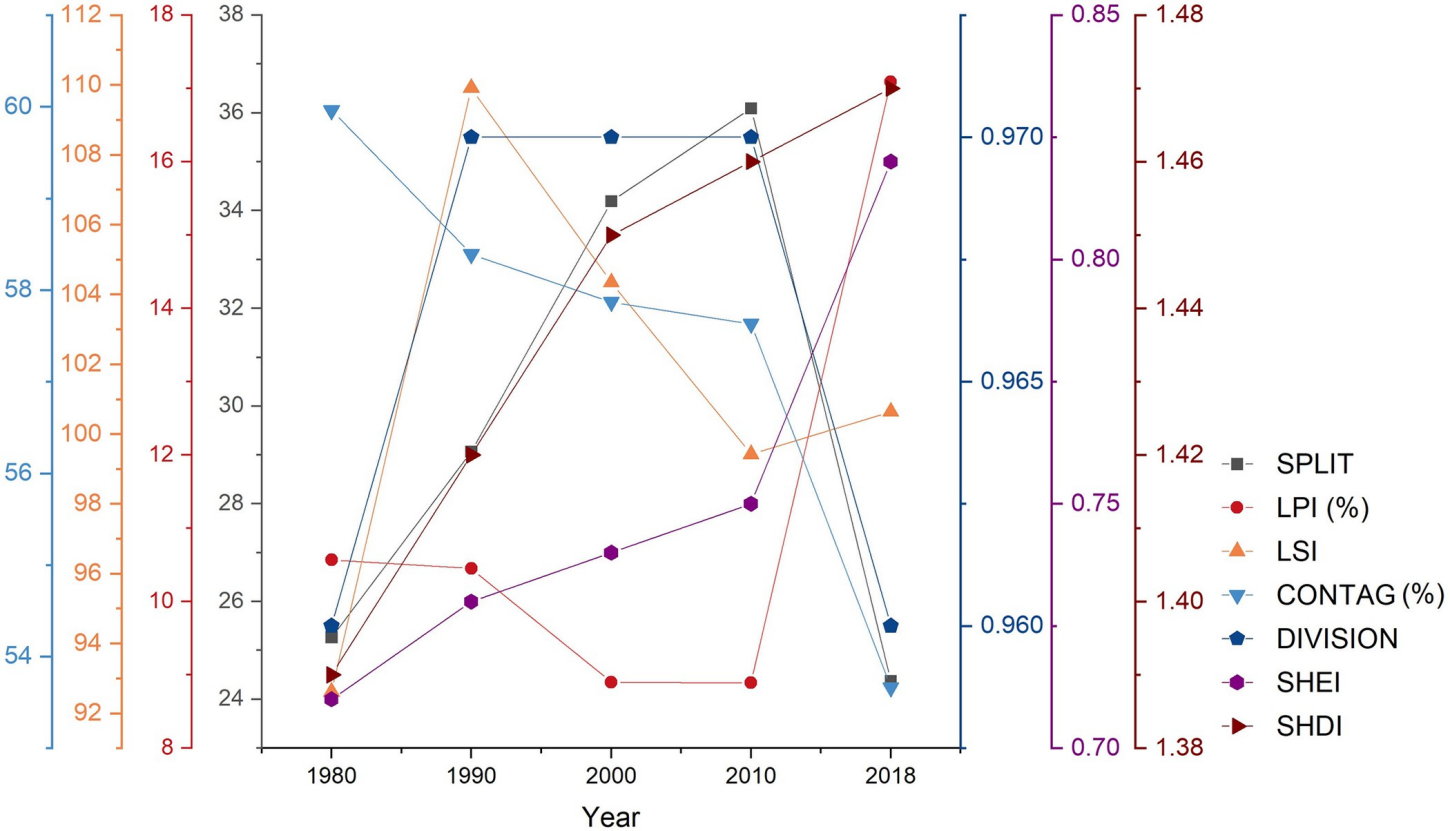
Changes in landscape pattern indices in the Beijing-Tianjin region from 1980 to 2018.

Changes in rural landscape fragmentation can be described by the SPLIT and LPI. From 1980 to 2010, the SPLIT of the natural rural landscape in the Beijing-Tianjin region continued to increase (Fig 10), and the LPI continued to decrease. This situation suggests that during these 30 years, the natural rural landscape patches were separated, leading to a reduction in the overall integrity of and a continuous increase in fragmentation. However, between 2010 and 2018, the SPLIT decreased significantly, dropping to levels lower than those in 1980. At the same time, the LPIs increased considerably, reaching higher levels than those in 1980. These results suggest that the fragmentation of the natural rural landscape in the Beijing-Tianjin region intensified from 1980 to 2010, reaching its most severe state in 2010, at which time it reversed.

Changes in rural landscape shape can be described by the LSI. Changes in the LSI indicate that the shape of the natural rural landscape in the Beijing-Tianjin region became more complex and irregular between 1980 and 1990 (Fig 10). The shape was restored between 1990 and 2010, as the landscape shape continuously changed in a more regular direction. After 2010, this positive trend reversed, and the landscape shape developed toward complexity and irregularity.

Changes in rural landscape connectivity can be described by CONTAG. The CONTAG of the natural rural landscape in the Beijing-Tianjin region decreased continuously between 1980 and 2010, followed by a slight increase between 2010 and 2018 (Fig 10). The ecological significance of this change is that between 1980 and 2010, as the degree of fragmentation of the natural landscape intensified, the dispersion and isolation of natural landscape patches increased, resulting in a gradual decline in landscape connectivity. Between 2010 and 2018, the slight increase in CONTAG indicated that the degree of connection between natural landscape patches increased, enhancing landscape connectivity and restoring overall integrity.

Changes in rural landscape separation can be described by DIVISION. The changes in DIVISION indicate that between 1980 and 1990 (Fig 10), the natural rural landscape patches in the Beijing-Tianjin region were separated, the degree of landscape separation increased, and the concentration decreased. Between 2010 and 2018, the degree of separation of natural rural landscape patches in the Beijing-Tianjin region decreased, their concentration increased, and landscape restoration achieved better results.

Changes in rural landscape heterogeneity can be described by the SHDI and SHEI. Between 1980 and 2018, the SHDI and SHEI of the natural rural landscape in the Beijing-Tianjin region continuously increased (Fig 10), with the SHDI growing faster between 1980 and 2000 and the SHEI growing faster between 2010 and 2018. The ecological significance of these changes is that between 1980 and 2018, the number and types of natural rural landscape patches in the Beijing-Tianjin region increased, and the patch distribution trended toward more balanced development, with the trend being more significant in the first 30 years. According to the previous analysis of the scale of natural landscape types, the dominant natural landscape type in the Beijing-Tianjin region has always been farmland; the increase in the SHEI meant that the dominance of farmland was weakening, with various natural landscapes in the region developing toward a more even distribution.

#### Comparative analysis of rural landscape and urban area patterns

Because rural landscapes are converted mainly into urban areas, analyzing the landscape patterns of urban areas and exploring the relationships between changes in urban areas and rural landscapes are highly important for understanding the evolution of rural landscapes [73,74].

According to the changes in landscape indices (Fig 10), urban areas expanded rapidly between 1980 and 2018, with a gradual increase in patch area. The degree of patch aggregation and cohesion maintained an increasing trend, particularly between 1990 and 2018. The connectivity and cohesion of urban areas generally maintained an increasing trend, with landscape patches tending toward networked structures. The shape of the urban areas became more complex and irregular between 1980 and 1990, but this trend was alleviated after 1990, with landscape patch shapes gradually tending toward regularization.

Farmland is the largest transferred rural landscape. A comparison of the processes underlying changes in urban areas and farmland shows that the scale of urban areas continued to expand, fragmentation continued to intensify, and urban shapes became complex and irregular. However, the scale of farmland continued to shrink, fragmentation continued to intensify, and farmland shapes became complex and irregular. The two landscapes were strongly correlated in terms of scale changes (Fig 6), fragmentation changes, and shape changes, which further supports the conclusion drawn earlier that during the rapid process of urbanization, the conversion of rural landscape types in the Beijing-Tianjin region involved mainly the conversion of farmland to urban areas. However, in terms of network changes, there were slight differences. The degree of network connectivity and patch concentration in urban areas has consistently increased over the past 40 years, while the degree of network connectivity in farmlands has not changed significantly; farmland patches have always been distributed in a relatively dispersed manner. This difference indicates that during rapid urbanization, human activities greatly impact urban areas; however, the importance of farmland is neglected. Furthermore, the ecological restoration and scientific planning of farmland following its encroachment and division during the conversion to urban and rural residential areas are ignored.

A comparison of the concentration changes in urban areas and overall landscape patterns in the Beijing-Tianjin region from 1980 to 2018 reveals that the concentration of natural rural landscapes continuously decreased between 1980 and 2010, reaching its most severe state in 2010, after which the degree of separation decreased and the concentration increased (Fig 10). However, the concentration of urban areas continuously increased from 1980 to 2018. The results indicate that the transfer, dispersion, and integration of natural rural landscape patches were passive during rapid urbanization. During the rapid development of urban areas, the number and density of patches fluctuated, but the concentration was continuously strengthened, indicating that the expansion of urban areas occured mainly by gradually spreading from the core of urban areas and rural residential areas to the surrounding areas, resulting in the erosion of the surrounding natural landscape and an increase in the degree of natural landscape fragmentation.

From 1980 to 2010, the fragmentation of natural rural landscapes intensified, leading to increased dispersion and disconnection of landscape patches and a gradual decline in connectivity. However, from 2010 to 2018, the integration of natural rural landscape patches improved, and connectivity increased. Moreover, the connectivity of the urban areas continuously strengthened from 1980 to 2018. A high degree of network connectivity is crucial for the development of urban areas because it can enhance the efficient flow of materials and information between regions. A comparison of the changes in connectivity between urban areas and natural rural landscapes reveals that new urban areas and transportation infrastructure projects enhanced the network connectivity of urban areas, whereas the connectivity of natural rural landscapes did not effectively improve. Some transportation infrastructure projects even fragmented natural rural landscape patches without subsequently facilitating ecological restoration and consolidation of these patches.

The shape of natural rural landscapes rapidly became more complex and irregular from 1980 to 1990, continually shifted toward regularity from 1990 to 2010, and became complex and irregular once again after 2010. However, the shape of the urban areas became complex and irregular from 1980 to 1990 and gradually became regularized after 1990. The authors identified two reasons for the contradictory changes in the shape of urban areas and natural rural landscapes from 2010 to 2018. First, human activities associated with the development of urban areas, which are more proactive, do not consider damage to natural rural landscapes or implement effective ecological restoration and scientific planning. Second, increased public awareness of ecological and environmental protection, along with policy guidance and public initiatives, such as returning farmland to forestland and grassland, ecological restoration, agricultural structure adjustments, and the cultivation of economic forests after land outsourcing, has led to a greater frequency of transfers between different types of natural rural landscapes. In the first situation, human intervention should be strengthened, and scientific planning and design should be used to achieve rapid and efficient restoration of rural landscapes. In the second situation, the government needs to reinforce top-level design and implement policy guidance for the transfer of rural landscapes, enabling landscapes within the region to achieve greater ecological benefits.

## Discussion

### Landscape pattern and drivers

The high spatial differentiation of rural landscape types in the Beijing-Tianjin region is not well balanced overall. In terms of elemental dimensions, the fragmentation of water bodies is the most severe, as a significant amount of farmland has been transformed into urban areas. The overall landscape pattern has gradually become fragmented, disjointed, and irregular. This can process leads to a decrease in biodiversity, a weakening of ecosystem service functions, and poorer habitat connectivity [75]. Currently, natural rural landscapes in the Beijing-Tianjin region remain at a relatively primitive stage and have not yet achieved a balanced design via regional planning to enhance regional landscape quality.

From 1980 to 2018, during rapid urbanization, the urban area of the Beijing-Tianjin region increased by 84%, and the permanent population increased from 16.497 million to 37.132 million, a 1.25-fold increase. At the same time, the GDP grew from 37.94 billion CNY to 5,418.09 billion CNY, a 141-fold increase. Rapid urbanization and population growth led cities to encroach on rural landscapes. The "economy-centered" development concept prioritized economic development as the government’s primary task, neglecting the integrity of rural landscapes and the creation of unique rural features. Cities acquired more land for the development of industries such as manufacturing and services through expansion, and even some rural areas saw the emergence of light industrial manufacturing enterprises [76]. Consequently, the growing demand for economic activities led to the gradual expansion of cities and the occupation of vast rural landscape areas. New urban areas and transportation infrastructure projects fragmented natural landscape patches without subsequently facilitating ecological restoration or consolidation of these patches through planning.

### Comparison with other rapidly urbanizing areas

Comparatively, research on land use and landscape pattern changes in Changchun, a provincial capital city in northern China, also indicates that rapid urbanization has led to the encroachment of substantial agricultural lands by urban expansion, resulting in the fragmentation of urban green spaces. Through urban planning interventions, Changchun subsequently integrated patches of urban green spaces into its core areas, restoring parts of the urban green space system [38]. Research by Wang et al. [77] on the structure of farmland in China indicates that urbanization and ecological restoration programs have been the main drivers of farmland loss. Farmland decreased by 5.92 million ha or 3.31% in China from 2000 to 2010 [77]. Studies on landscape pattern changes in the Yangtze River Basin have shown that the dynamic attitude of farmland continues to decrease, while the dynamic state of urban areas continues to increase [45]. Moreover, the degree of landscape fragmentation in the Yangtze River basin has been increasing with urbanization. Assessments of landscape characteristics in the Douro vineyard region show that during the process of urbanization, from 1995 to 2015, the urban area increased by 38.8%, while the farmland decreased by 2.1% [44]. Human activities are the main drivers behind changes in land use and landscape patterns. These conclusions are similar to those of this study, indicating that the changes in landscape patterns observed in the Beijing-Tianjin region are part of a broader phenomenon observed in other rapidly urbanizing areas. However, the changes in landscape patterns in the Beijing-Tianjin region also exhibit some unique characteristics, such as varying degrees of impact on each type of natural landscape due to expansion. The rate of change in natural landscapes in the Beijing-Tianjin region exceeds that in the Yangtze River Basin and Changchun. The overall landscape pattern in the Beijing-Tianjin region has undergone significant fragmentation compared to that in other regions. These unique features are attributed to the distinct geographical conditions and capital region positioning of the Beijing-Tianjin region.

### Policy and planning recommendations

According to the spatial features of the four types of rural landscapes, future ecological protection and restoration efforts should focus on maintaining the area and integrity of farmland, enhancing the continuity and network connectivity of water bodies, and increasing the area and distribution uniformity of forestland and grassland.

During the urbanization process, it is essential to scientifically control the form of urban expansion, preventing the disorderly encroachment of rural lands, with particular emphasis on the protection of forestlands, grasslands, water bodies, and other natural landscapes. Government planning and supervisory departments should monitor landscape patterns in real time, promptly intervening to halt land use practices that disrupt the overall landscape pattern and ecological environment. Specifically, the overall regional landscape pattern should be systemically assessed, and the fragmentation, dispersion, heterogeneity, and irregularity of rural landscapes should be addressed. The proactive integration and restoration of rural landscapes should be incorporated into planning along with improvements in ecological protection and compensation mechanisms, regulations, and policies. Regular quantitative assessments of rural landscape patterns should be conducted to promptly identify issues and supplement rural landscapes. The ecological performance accountability system should be improved, with higher-level regulatory agencies holding regions with substandard ecological performance accountable and urging corrections [78,79]. An ecological control planning system that focuses on ecological objectives in regional planning, addresses regional ecological environmental issues with specific planning schemes, and enhances the quality of rural landscapes should be established and continuously improved.

Although the traditional landscape image of northern Chinese plain villages is "village + field", the Beijing-Tianjin region also boasts abundant forest, grassland, and water bodies. Currently, these three types of rural landscapes have not been organically integrated into rural settlements to form beautiful and harmonious rural scenery. In future urban areas and rural planning, to create distinctive rural landscapes, rural networks based on geographically close villages with multifunctional rural areas and well-connected elements should be used as basic units. Various types of natural elements should be integrated and distributed relatively evenly within rural networks, ultimately leading to the formation of organic combinations of rural settlements and natural elements and improving the quality of rural living environments.

Additionally, the rural landscape of the Beijing-Tianjin region represents the agrarian civilization characteristic of Chinese culture, and preserving traditional rural landscapes is highly important for the continuation of regional culture. Establishing a management system for rural landscapes that continues cultural traditions is crucial for natural rural landscapes imbued with historical memories, such as the Beijing-Hangzhou Grand Canal and other significant natural and cultural heritages. In planning and design, their cultural connotations and characteristics should be considered to continue the transmission of cultural heritage and thereby preserve a healthy ecological foundation and profound cultural roots for human society.

The southeastern part of the Beijing-Tianjin region boasts a wealth of water bodies, which tend to form dense networks with extensive areas of influence, thereby contributing to the enhancement of neighboring rural landscapes and improvements in ecological and environmental quality. During the process of guiding rural landscape features in areas rich in water resources, restoration efforts should focus on natural water bodies, strengthen their continuity and network connectivity, and reduce fragmentation. Water bodies can serve as ecological corridors, connecting different rural network units and enhancing regional rural coordination. For lakes, rivers, and canals, which are crucial aquatic landscape nodes, their banks should undergo ecological restoration and landscape planning to foster diverse and indigenous water bodies that facilitate landscape enhancement, economic and trade exchanges, element flow, and the inheritance of regional historical culture. While urban areas and rural construction activities continue, attention should be given to preserving the area and quality of water bodies, coordinating the relationship between villages and surrounding natural rural landscapes, and shaping a northern town landscape where water and fields blend.

### Limitations and future research

The limitations of this study are primarily due to data accessibility constraints, necessitating reliance on remote sensing imagery with a resolution of 30*30 m. This constraint might introduce some inaccuracies, particularly when analyzing land use in smaller rural locales. While this resolution is widely adopted for broad landscape pattern analyses, employing higher-resolution remote sensing imagery in future studies could significantly enrich the findings.

An additional limitation lies in the use of landscape index analysis, which can delineate differences and transformations in landscape patterns only through comparative index analysis; it does not enable the level of a landscape pattern to be directly inferred from a singular index. Future research could explore the development of a comprehensive landscape index measurement framework, which would simplify the assessment of landscape pattern characteristics for researchers and aid government planning departments in real-time monitoring of landscape pattern quality.

Moreover, in this study, the causal relationship behind urban expansion-induced rural landscape pattern alterations was approached from demographic growth and socioeconomic perspectives without engaging in a quantitative correlation analysis. Future investigations could segment the Beijing-Tianjin region into zones based on economic development levels and quantitatively dissect the driving factors behind rural landscape pattern shifts, yielding more nuanced insights. Additionally, the potential direct impacts of landscape pattern modifications on the ecological environment warrant further exploration. Future studies could quantitatively evaluate how changes in landscape patterns affect ecosystem services, environmental quality, biodiversity, ecological resilience, and ecological networks, thereby providing a holistic understanding of their ecological implications.

## Conclusions

In this study, a quantitative approach is employed, leveraging spatial pattern analysis and landscape index analysis, to meticulously investigate the transformation of rural landscapes in the Beijing-Tianjin region, drawing on LUCC data spanning from 1980 to 2018. The conclusions are as follows: (1) There is significant spatial differentiation in rural landscapes in the Beijing-Tianjin region; farmland and forestland are the main types. Forestland and grassland are concentrated in the northwestern mountainous areas, whereas water bodies originate from the mountains, flow through central villages and farmland, and converge in the southeast to form a river system that eventually flows into the Bohai Sea, creating a "mountain-field-sea" natural landscape pattern. (2) Accompanied by rapid urbanization, the conversion of rural landscapes in the Beijing-Tianjin region mainly involves the conversion of farmland to urban areas, with few exchanges between other landscape types. The analysis indicated a 23% reduction in farmland area and a 2% expansion in urban areas throughout the duration of the study. Furthermore, there was a 2% growth in forestland and a 4% increase in grassland during the same period. With rapid urbanization, the urban areas in the Beijing-Tianjin region increased by 3% per decade, while farmland decreased at the same rate. Urban areas expanded in a circular pattern from urban area centers and rural residential areas, gradually forming a networked structure. However, farmland shrank, and contiguous farmland became fragmented. (3) With rapid urbanization, the elements of rural landscapes in the Beijing-Tianjin region experienced a reduction in scale, intensified fragmentation, and continuous shape fluctuations. Water bodies exhibited the highest level of fragmentation among landscapes, with the NP increasing by 36% during the study period. Additionally, water bodies demonstrated significant network connectivity. Conversely, forestlands had the strongest concentration of all the landscape types. Concurrently, the rural landscape patterns in the Beijing-Tianjin region tended toward fragmentation, dispersion, and heterogeneity, with the shape undergoing a transition from complexity to regularity. Passive changes in rural landscape patches in the Beijing-Tianjin region, including changes in transfer, dispersion, and integration, have not been effectively restored or scientifically planned after damage.

## Supporting information

S1 Table The landscape indices of various landscape types in the Beijing-Tianjin region in 2018 at the class level. (DOCX)

S2 Table Area of various rural landscape types in Beijing and Tianjin from 1980 to 2018. (DOCX)

S3 Table Landscape convert matrix in Beijing-Tianjin region from 1980 to 1990. (DOCX)

S4 Table Landscape convert matrix in Beijing-Tianjin region from 1990 to 2000. (DOCX)

S5 Table Landscape convert matrix in Beijing-Tianjin region from 2000 to 2010. (DOCX)

S6 Table Landscape convert matrix in Beijing-Tianjin region from 2010 to 2018. (DOCX)

S7 Table Landscape pattern indices at class metrics in Beijing-Tianjin region from 1980 to 2018. (DOCX)

S8 Table Landscape pattern indices change at landscape metrics in Beijing-Tianjin region from 1980 to 2018. (DOCX)
