# Supplementary material for: The spatiotemporal evolution of rural landscape patterns in Chinese metropolises under rapid urbanization
Source: PLoS One. 2024 May 6;19(5):e0301754. doi: 10.1371/journal.pone.0301754 (PMC11073728; doi:10.1371/journal.pone.0301754)
Supplement: S1 Table — (DOCX) [file pone.0301754.s001.docx]

**S1 Table**

| Landscape | CA | PLAND | NP | PD | LPI | AREA_MN | AI | COHESION | FRAC_MN |
| --- | --- | --- | --- | --- | --- | --- | --- | --- | --- |
| Farmland | 965494.98 | 34.01 | 1401.00 | 0.05 | 4.86 | 689.15 | 96.71 | 99.82 | 1.12 |
| Forestland | 799334.91 | 28.16 | 1367.00 | 0.05 | 17.09 | 584.74 | 98.11 | 99.91 | 1.08 |
| Grassland | 160255.26 | 5.65 | 1452.00 | 0.05 | 0.17 | 110.37 | 93.11 | 98.55 | 1.12 |
| Water body | 220039.56 | 7.75 | 3144.00 | 0.11 | 4.17 | 69.99 | 93.74 | 99.84 | 1.07 |
| Urban area | 662188.68 | 23.33 | 9304.00 | 0.33 | 4.36 | 71.17 | 96.03 | 99.16 | 1.06 |
| Unused land | 31310.01 | 1.10 | 141.00 | 0.01 | 0.46 | 222.06 | 97.00 | 99.18 | 1.10 |
