# Supplementary material for: The spatiotemporal evolution of rural landscape patterns in Chinese metropolises under rapid urbanization
Source: PLoS One. 2024 May 6;19(5):e0301754. doi: 10.1371/journal.pone.0301754 (PMC11073728; doi:10.1371/journal.pone.0301754)
Supplement: S2 Table — (DOCX) [file pone.0301754.s002.docx]

**S2 Table**

| Landscape | 1980 | | 1990 | | 2000 | | 2010 | | 2018 | |
| --- | --- | --- | --- | --- | --- | --- | --- | --- | --- | --- |
|  | Area  （km²） | Proportion | Area  （km²） | Proportion | Area  （km²） | Proportion | Area  （km²） | Proportion | Area  （km²） | Proportion |
| Farmland | 12572.95 | 0.45 | 11895.63 | 0.42 | 10967.76 | 0.39 | 10159.06 | 0.36 | 9661.95 | 0.34 |
| Forestland | 7831.42 | 0.28 | 7785.04 | 0.27 | 7845.22 | 0.28 | 7849.48 | 0.28 | 7994.19 | 0.28 |
| Grassland | 1546.21 | 0.06 | 1540.28 | 0.05 | 1474.92 | 0.05 | 1556.74 | 0.05 | 1602.58 | 0.06 |
| Water body | 2236.33 | 0.08 | 2213.50 | 0.08 | 2338.35 | 0.08 | 2158.75 | 0.08 | 2198.94 | 0.08 |
| Urban area | 3592.53 | 0.13 | 4672.71 | 0.16 | 5471.84 | 0.19 | 6352.77 | 0.22 | 6615.55 | 0.23 |
| Unused land | 99.88 | 0.00 | 256.31 | 0.01 | 265.73 | 0.01 | 296.60 | 0.01 | 313.04 | 0.01 |
