# Supplementary material for: The spatiotemporal evolution of rural landscape patterns in Chinese metropolises under rapid urbanization
Source: PLoS One. 2024 May 6;19(5):e0301754. doi: 10.1371/journal.pone.0301754 (PMC11073728; doi:10.1371/journal.pone.0301754)
Supplement: S3 Table — (DOCX) [file pone.0301754.s003.docx]

**S3 Table**

| Landscape | Farmland | Forestland | Grassland | | Water body | Urban area | Unused land | Total |
| --- | --- | --- | --- | --- | --- | --- | --- | --- |
| Farmland | 11083.58 | 81.35 | | 56.85 | 435.80 | 903.67 | 10.84 | 12572.09 |
| Forestland | 98.96 | 7684.40 | | 10.69 | 5.21 | 31.89 | 0.00 | 7831.15 |
| Grassland | 67.65 | 2.83 | | 1387.32 | 34.31 | 48.26 | 5.77 | 1546.14 |
| Water body | 226.37 | 8.07 | | 60.31 | 1451.57 | 269.55 | 198.77 | 2214.64 |
| Urban area | 292.27 | 4.24 | | 11.62 | 47.10 | 3228.66 | 8.53 | 3592.42 |
| Unused land | 9.01 | 0.01 | | 6.48 | 42.17 | 16.56 | 25.24 | 99.47 |
| Total | 11777.84 | 7780.9 | | 1533.27 | 2016.16 | 4498.59 | 249.15 | 27855.91 |
