# Supplementary material for: The spatiotemporal evolution of rural landscape patterns in Chinese metropolises under rapid urbanization
Source: PLoS One. 2024 May 6;19(5):e0301754. doi: 10.1371/journal.pone.0301754 (PMC11073728; doi:10.1371/journal.pone.0301754)
Supplement: S4 Table — (DOCX) [file pone.0301754.s004.docx]

**S4 Table**

| Landscape | Farmland | Forestland | Grassland | | Waterbody | Urban area | Unused land | Total |
| --- | --- | --- | --- | --- | --- | --- | --- | --- |
| Farmland | 10883.70 | 73.88 | | 26.07 | 163.70 | 747.11 | 1.06 | 11895.53 |
| Forestland | 16.74 | 7735.86 | | 15.01 | 1.68 | 15.70 | 0.00 | 7785.00 |
| Grassland | 28.30 | 25.65 | | 1426.27 | 34.03 | 25.99 | 0.02 | 1540.26 |
| Water body | 21.39 | 7.79 | | 5.90 | 2118.86 | 37.37 | 20.92 | 2212.23 |
| Urban area | 17.59 | 2.03 | | 1.49 | 12.10 | 4638.81 | 0.59 | 4672.61 |
| Unused land | 0.02 | 0.00 | | 0.17 | 7.33 | 5.61 | 243.14 | 256.28 |
| Total | 10967.74 | 7845.22 | | 1474.92 | 2337.7 | 5470.59 | 265.73 | 28361.91 |
