# Supplementary material for: The spatiotemporal evolution of rural landscape patterns in Chinese metropolises under rapid urbanization
Source: PLoS One. 2024 May 6;19(5):e0301754. doi: 10.1371/journal.pone.0301754 (PMC11073728; doi:10.1371/journal.pone.0301754)
Supplement: S5 Table — (DOCX) [file pone.0301754.s005.docx]

**S5 Table**

| Landscape | Farmland | Forestland | Grassland | | Water body | Urban area | Unused land | Total |
| --- | --- | --- | --- | --- | --- | --- | --- | --- |
| Farmland | 10029.2 | 22.1 | | 41.5 | 83.5 | 788.7 | 2.5 | 10967.6 |
| Forestland | 5.5 | 7808.8 | | 4.1 | 0.6 | 26.3 | 0 | 7845.2 |
| Grassland | 2.4 | 1.7 | | 1457.8 | 5.3 | 7.7 | 0 | 1474.9 |
| Water body | 90.9 | 12.5 | | 43.7 | 2059.9 | 92.8 | 38.2 | 2338 |
| Urban area | 29.6 | 3 | | 8.4 | 5.8 | 5425 | 0 | 5471.8 |
| Unused land | 1.5 | 0 | | 1.3 | 1.8 | 5.3 | 255.8 | 265.7 |
| Total | 10159 | 7848.1 | | 1556.7 | 2156.8 | 6345.9 | 296.6 | 28363.2 |
