# Supplementary material for: The spatiotemporal evolution of rural landscape patterns in Chinese metropolises under rapid urbanization
Source: PLoS One. 2024 May 6;19(5):e0301754. doi: 10.1371/journal.pone.0301754 (PMC11073728; doi:10.1371/journal.pone.0301754)
Supplement: S6 Table — (DOCX) [file pone.0301754.s006.docx]

**S6 Table**

| Landscape | Farmland | Forestland | Grassland | | Water body | Urban area | Unused land | Total |
| --- | --- | --- | --- | --- | --- | --- | --- | --- |
| Farmland | 9601.01 | 150.23 | | 28.91 | 49.85 | 320.21 | 8.65 | 10158.86 |
| Forestland | 6.07 | 7799.56 | | 13.58 | 7.76 | 21.84 | 0.50 | 7849.31 |
| Grassland | 1.70 | 14.34 | | 1509.84 | 11.28 | 19.44 | 0.11 | 1556.71 |
| Water body | 12.43 | 3.81 | | 7.96 | 2084.69 | 32.37 | 17.46 | 2158.72 |
| Urban area | 40.54 | 26.07 | | 40.00 | 23.03 | 6218.54 | 4.54 | 6352.72 |
| Unused land | 0.05 | 0.01 | | 2.23 | 11.96 | 0.57 | 281.77 | 296.59 |
| Total | 9661.79 | 7994.02 | | 1602.52 | 2188.57 | 6612.97 | 313.04 | 28372.91 |
