# Supplementary material for: The spatiotemporal evolution of rural landscape patterns in Chinese metropolises under rapid urbanization
Source: PLoS One. 2024 May 6;19(5):e0301754. doi: 10.1371/journal.pone.0301754 (PMC11073728; doi:10.1371/journal.pone.0301754)
Supplement: S7 Table — (DOCX) [file pone.0301754.s007.docx]

**S7 Table**

| Landscape | Year | CA | PLAND | NP | PD | LPI | AREA_MN | AI | COHESION | FRAC_MN |
| --- | --- | --- | --- | --- | --- | --- | --- | --- | --- | --- |
| Farmland | 1980 | 1256266.71 | 44.84 | 1712.00 | 0.06 | 9.23 | 733.80 | 97.39 | 99.90 | 1.09 |
|  | 1990 | 1188199.53 | 41.86 | 2093.00 | 0.07 | 7.74 | 567.70 | 96.77 | 99.90 | 1.09 |
|  | 2000 | 1095769.44 | 38.60 | 2004.00 | 0.07 | 6.67 | 546.79 | 96.79 | 99.86 | 1.09 |
|  | 2010 | 1015067.52 | 35.76 | 1420.00 | 0.05 | 6.11 | 714.84 | 96.79 | 99.84 | 1.11 |
|  | 2018 | 965494.98 | 34.01 | 1401.00 | 0.05 | 4.86 | 689.15 | 96.71 | 99.82 | 1.12 |
| Forestland | 1980 | 783070.11 | 27.95 | 2266.00 | 0.08 | 10.57 | 345.57 | 98.12 | 99.89 | 1.07 |
|  | 1990 | 778426.47 | 27.42 | 2278.00 | 0.08 | 10.45 | 341.71 | 98.11 | 99.89 | 1.07 |
|  | 2000 | 784437.93 | 27.63 | 1491.00 | 0.05 | 8.90 | 526.12 | 98.15 | 99.88 | 1.08 |
|  | 2010 | 784858.50 | 27.65 | 1197.00 | 0.04 | 8.89 | 655.69 | 98.19 | 99.88 | 1.09 |
|  | 2018 | 799334.91 | 28.16 | 1367.00 | 0.05 | 17.09 | 584.74 | 98.11 | 99.91 | 1.08 |
| Grassland | 1980 | 154645.47 | 5.52 | 2084.00 | 0.07 | 0.13 | 74.21 | 93.33 | 98.56 | 1.09 |
|  | 1990 | 154043.73 | 5.43 | 2290.00 | 0.08 | 0.13 | 67.27 | 92.94 | 98.48 | 1.09 |
|  | 2000 | 147515.58 | 5.20 | 1821.00 | 0.06 | 0.13 | 81.01 | 92.96 | 98.49 | 1.10 |
|  | 2010 | 155666.25 | 5.48 | 1427.00 | 0.05 | 0.18 | 109.09 | 93.20 | 98.56 | 1.11 |
|  | 2018 | 160255.26 | 5.65 | 1452.00 | 0.05 | 0.17 | 110.37 | 93.11 | 98.55 | 1.12 |
| Water body | 1980 | 223683.30 | 7.98 | 2308.00 | 0.08 | 5.27 | 96.92 | 96.27 | 99.81 | 1.07 |
|  | 1990 | 221544.45 | 7.80 | 4926.00 | 0.17 | 2.18 | 44.97 | 94.04 | 99.66 | 1.07 |
|  | 2000 | 233962.74 | 8.24 | 3626.00 | 0.13 | 2.26 | 64.52 | 94.24 | 99.69 | 1.07 |
|  | 2010 | 216010.62 | 7.61 | 3224.00 | 0.11 | 2.03 | 67.00 | 93.78 | 99.75 | 1.07 |
|  | 2018 | 220039.56 | 7.75 | 3144.00 | 0.11 | 4.17 | 69.99 | 93.74 | 99.84 | 1.07 |
| Urban area | 1980 | 360271.53 | 12.86 | 14220.00 | 0.51 | 1.11 | 25.34 | 1.06 | 97.88 | 93.41 |
|  | 1990 | 468486.81 | 16.50 | 17642.00 | 0.62 | 1.50 | 26.56 | 1.06 | 98.54 | 93.60 |
|  | 2000 | 548111.97 | 19.31 | 13132.00 | 0.46 | 3.81 | 41.74 | 1.06 | 99.16 | 94.89 |
|  | 2010 | 636067.53 | 22.41 | 10791.00 | 0.38 | 4.69 | 58.94 | 1.06 | 99.23 | 95.93 |
|  | 2018 | 662188.68 | 23.33 | 9304.00 | 0.33 | 4.36 | 71.17 | 1.06 | 99.16 | 96.03 |
