# Supplementary material for: The spatiotemporal evolution of rural landscape patterns in Chinese metropolises under rapid urbanization
Source: PLoS One. 2024 May 6;19(5):e0301754. doi: 10.1371/journal.pone.0301754 (PMC11073728; doi:10.1371/journal.pone.0301754)
Supplement: S8 Table — (DOCX) [file pone.0301754.s008.docx]

**S8 Table 8**

| Year | SPLIT | LPI | LSI | CONTAG | DIVISION | SHEI | SHDI |
| --- | --- | --- | --- | --- | --- | --- | --- |
| 1980 | 25.27 | 10.57 | 92.61 | 59.96 | 0.96 | 0.71 | 1.39 |
| 1990 | 29.07 | 10.45 | 109.91 | 58.39 | 0.97 | 0.73 | 1.42 |
| 2000 | 34.19 | 8.90 | 104.34 | 57.87 | 0.97 | 0.74 | 1.45 |
| 2010 | 36.09 | 8.89 | 99.42 | 57.63 | 0.97 | 0.75 | 1.46 |
| 2018 | 24.37 | 17.09 | 100.64 | 53.66 | 0.96 | 0.82 | 1.47 |
